# Moyamoya Disease: A Review of Current Literature

**DOI:** 10.7759/cureus.10141

**Published:** 2020-08-30

**Authors:** Apurv Gupta, Anshika Tyagi, Moises Romo, Krystal C Amoroso, FNU Sonia

**Affiliations:** 1 Department of Surgery, Maulana Azad Medical College, New Delhi, IND; 2 Department of Medicine and Nutrition, University of Guanajuato, Leon, MEX; 3 Department of Medicine, University of West Indies, St. Augustine, TTO; 4 Department of Medicine, Albert Einstein College of Medicine, Bronx, USA

**Keywords:** moyamoya disease, arteriopathy, cerebral angiography, rnf213, revascularization, recurrent strokes, hyperperfusion syndrome, cilostazol, brush sign, ivy sign

## Abstract

Moyamoya disease (MMD) is an infrequent disease of cerebral vasculature characterized by long-standing and progressive occlusion of large intracranial arteries. It is seen predominantly in the East Asian population. Most of the cases of MMD are sporadic, but there is a small percentage that is familial. The mode of inheritance is reported to be autosomal dominant with incomplete penetrance. Studies show that the susceptibility gene of MMD is located on chromosome 17. The clinical presentation is variable and is influenced by the age and geographic region of the patient. Children mainly present with ischemia-related neurologic episodes whereas MMD in adults can manifest as either an ischemic event or an intracranial hemorrhage (ICH). The gold standard investigation for diagnosis is cerebral angiography which reveals a smoky appearance of arteries at the base of the skull, thus granting the disease its name. The treatment is mostly surgical and includes direct and indirect revascularization procedures, which prevent the recurrence of both ischemic and hemorrhagic strokes. However, combination revascularization procedures are now on the rise due to studies showing better long-term outcomes. The aim of the article is to critically analyze the current literature and updates on various aspects of MMD including, but not limited to, etiology, diagnosis, and treatment.

## Introduction and background

Moyamoya disease (MMD) is a rare disease found with varying frequency in different geographical regions. It is characterized by chronic and progressive stenosis of the distal internal carotid artery (ICA) and its tributaries, anterior carotid artery (ACA) and the middle carotid artery (MCA). It gets its name from the hazy smoke-like vessels formed in the base of the skull as seen on digital subtraction angiography [1]. The highest number of cases are seen in East Asia, with more women affected than men. Based on epidemiological studies, the incidence of MMD is 0.94 and 2.3 per 100,000 individuals in Japan and South Korea, respectively [1]. While the majority of cases are sporadic, around 10%-15% of patients have a family history of MMD. Although etiology is unestablished, studies point to the involvement of chromosome 17 [2]. Outside of East Asia, the incidence in regions like North America can drop to 0.09/100,000 individuals, although a rising trend has been noticed recently [1]. MMD maintains a classical pattern of bimodal age distribution. The first peak occurs around the age of five and the second one around the age of 40. An ischemic profile is predominant in the pediatric age group. In adults, both ischemia and intracranial hemorrhage (ICH) are possible [3].

Diagnosis is formed with the use of cerebral angiography. Non-invasive imaging includes computed tomography (CT) angiography and magnetic resonance angiography (MRA) [4]. Earlier, bilateral involvement was a mandatory criterion for the diagnosis of MMD. With the increased finding of MMD in patients with unilateral occlusion, diagnostic criteria were revised to include a unilateral presentation as well [4]. Treatment largely revolves around surgical interventions. Direct and indirect revascularization surgeries are performed to improve blood circulation in the affected region [5]. This article will explore the developments in our understanding of the disease and its treatment, based on the existing literature.

## Review

When the disease is present without any associated disorders, it is termed as MMD [6]. MMD, in presence of other acquired or familial conditions, is known as quasi-MMD. In comparison to MMD, unilateral involvement is more pronounced and ICH is less commonly documented in quasi-MMD [7].

Etiology

Despite recent studies on genetics in MMD, etiology remains largely unclear. Chromosome 17 has been identified as the carrier of the susceptibility gene of MMD, especially among people living in East Asia [4]. A comparative study noticed the presence of genetic anticipation and concluded that females were more affected in familial MMD [8]. One study also concluded that the mode of inheritance is autosomal dominant and penetrance is incomplete in familial MMD [9]. A genome-wide and locus-specific association study was done by Kamada et al. that discovered RNF213 as the first susceptibility gene in MMD [10]. They consequently documented p.R4859K to be the founder of missense mutation in RNF213. The higher carrier frequency of p.R4859K in specific geographical regions like Japan may explain the higher prevalence of MMD in East Asia as compared to Western countries. One case-control study also revealed that p.R4859K is linked with autoimmune and atherosclerotic quasi-MMD [11].

Pathogenesis

The direct effect of a mutation in RNF213 on the development of MMD and quasi-MMD remains to be elucidated. However, several mechanisms have been hypothesized. One such mechanism is that the presence of pro-inflammatory cytokines in addition to mutated RNF213 leads to increased angiogenesis [12]. The inability of mutated RNF213 to cause changes seen in MMD, without any added insults, was also indicated in an experiment. It was performed using mice with only p.R4828K missense mutation in RNF213. It was observed that there was no significant change in vessel diameter or vascular remodeling after common carotid artery ligation between mutated and wild-type mice [13]. Another study demonstrated a higher expression of matrix metalloproteinase (MMP)-9 in RNF213 knockout mice. Levels of MMP-9 are also increased in patients of MMD, thereby suggesting a possible association between them [14]. Nanba et al. detected an increased level of hepatocyte growth factor (HGF) in cerebrospinal fluid (CSF) and diseased arteries in patients with MMD. HGF is a potent inducer of angiogenesis and increased levels in patients indicate its likely role in the pathogenesis [15]. Elevation of basic fibroblast growth factor (bFGF) in CSF is documented in MMD and its levels may also help in predicting the status of angiogenesis post-revascularization [16]. The role of transforming growth factor-beta1 has been indicated in the pathogenesis as well [17]. One study also detected a raised number of circulating endothelial progenitor cells, which might explain the collateral vasculature in MMD [18].

Clinical features

MMD can have multiple presentations, partly influenced by age and geographical region [4,19]. There is a difference in clinical profiles in the United States, where there is a lower rate of intracranial bleeding in patients of MMD, as compared to Asia [19].

Ischemia

An ischemic event is the most common initial presentation. One study done on 528 patients in China reached to a conclusion that most cases of MMD present with ischemia. The hemorrhagic presentation was found more in adult patients. They further reported that ischemic type has a better prognosis and lower rate of rebleeding [20]. An ischemic event can manifest as either a transient ischemic attack (TIA) or ischemic stroke. The underlying cause is a vessel occlusion that leads to hypoperfusion of the cerebrum. Certain factors like hyperventilation, crying, and playing instruments such as harmonica or flute have been shown to exacerbate ischemic symptoms [4]. Anterior circulation is involved more commonly than posterior circulation [4].

The infarct pattern in MMD has been described to be different from a conventional stroke. A study was conducted on patients of MMD who presented with acute cerebral infarction. The atypical pattern on diffusion-weighted MRI (honeycombing, atypical territorial, and gyral) was observed more commonly than the typical pattern of stroke (deep lacunar, multiple-dots, borderzone, and territorial) [21]. The relationship between disease severity and site of the lesion has been explored as well. A study showed cortical involvement produced more severe disease than subcortical involvement [22]. Moreover, posterior stroke (affecting parieto-occipital) was associated with higher disease severity as compared to a lesion of anterior circulation (fronto-temporal) [22].

The presence of a variant of RNF213 genotype also influences disease severity. Homozygous presence of c.14576G>A variant in RNF213 is associated with an earlier age of onset of infarctions and involvement of posterior circulation (more severe disease) in comparison to heterozygotes [22,23]. This was further documented in a case report on siblings with homozygous and heterozygous expression of c.14576G>A variant in which the homozygote had an earlier onset of MMD with rapid disease progression [24]. Recurrent ischemic strokes can lead to complications in children such as developmental delay and mental retardation [25]. There is a case report on an Anglo-Saxon child with MMD, who presented predominantly with dementia [26]. 

Hemorrhage

ICH occurs with a higher frequency in adults than children [3]. It is even more frequent in adults greater than 40 years of age. Hemorrhagic MMD manifests most commonly as impaired consciousness [1]. Bleeding is noticed to occur more in anterior circulation dependant sites [4]. In a prospective cohort study, the presence of cerebral microbleeds (CMB) was observed to be a risk factor for intraventricular hemorrhage. The location of CMB also affected the outcome, for example, CMB located in the deep and periventricular white matter had more chances of subsequent ventricular bleed than CMB in any other location [27].

Other Features

MMD can present sometimes with seizures, likely to be a consequence of ischemia-induced damage [4]. In a study by Seol et al., significant headache was reported in approximately 21% of children [28]. It has been proposed that redistribution and decreased blood flow in the cerebrum lead to headaches in children [28]. Various involuntary movements can be seen in MMD, with chorea being the most typical of them. Decreased blood supply to basal ganglia-thalamocortical circuits is the proposed mechanism behind chorea [29].

Diagnosis

Cerebral angiography is the gold standard investigation [1]. As proposed by a Japanese research committee in 1997, definitive diagnosis of MMD requires the following on cerebral angiography: (1) occlusion at terminal ICA or proximal ACA/MCA, (2) abnormal network of vessels in proximity to the stenosed vessel, and (3) bilateral involvement [30]. In 2012, revisions were made to include MRA findings in the definitive diagnosis criteria. Cases with unilateral involvement were categorized as probable MMD [1]. MRA staging includes the following: (i) occlusion at terminal ICA or proximal ACA/MCA. (ii) presence of abnormal vessels in the basal ganglion, which can also be diagnosed by multiple flow voids on MRI of the brain. and (iii) bilateral angiography findings [31]. It is crucial to rule out other differential diagnoses - atherosclerosis, type 1 neurofibromatosis, meningitis, autoimmune diseases, traumatic brain injury, et cetera [31].

Angiographic staging of MMD, given by Suzuki, does not correlate with disease severity but explains the pathological changes seen over a period of time in each patient [32]. These stages demonstrate internal carotid - external carotid conversion quite well. Initial stages (stages 1-3) are characterized by occlusion of distal ICA with the development of moyamoya vessels. Late stages (stages 4-5) have anastomosis from the external carotid artery (ECA) followed by the fading of abnormal vessels. The last stage (stage 6) is marked by vanishing of ICA [32].

Supportive Findings

MRI: Non-specific signs like “ivy sign” and “brush sign” can be appreciated on MRI [33,34]. Ivy sign is characterized by diffuse leptomeningeal enhancement, as seen on fluid-attenuated inversion-recovery (FLAIR) or contrast-enhanced magnetic resonance images [35]. Brush sign is characterized by the increased visibility of deep medullary veins, seen best on susceptibility-weighted MRI [36].

Electroencephalography (EEG): High-voltage slow waves on EEG develop after the end of hyperventilation in children with MMD who present with cerebral ischemia. This is also known as the “rebuild-up” phenomenon [37].

Histopathology: Histopathological analysis of surgical specimens can help in supporting the diagnosis of MMD. Characteristic findings include thickened arterial intima of proximal ICA, thinning of the medial wall and waviness of internal elastic lamina of ACA/MCA/PCA, and perforating vascular channels around the circle of Willis [31].

Cerebral blood flow: Single Photon Emission Computed Tomography (CBF-SPECT) and Positron Emission Tomography (PET): CBF-SPECT and PET have been employed successfully in the evaluation of cerebral blood flow (CBF), which has major implications in diagnosing and staging the severity of cerebral ischemia/infarct in MMD (ischemic type) [31]. In addition, low CBF is also an indication for revascularization surgery [31].

Medical management

*Acute Phase*

Management of an acute case of ischemic stroke due to MMD is largely symptomatic [30]. Intravenous tissue plasminogen activator is contraindicated in ischemic-type MMD [31]. Aspirin should be given to children with acute anterior ischemic stroke due to MMD [36]. In the hemorrhagic presentation, anti-platelet and anti-coagulation drugs should be immediately stopped and antihypertensive therapy considered if the blood pressure is equal to or greater than 180/105 [31].

Chronic Phase

Aspirin is recommended for secondary prevention of stroke but the first priority is to evaluate the patient for surgical revascularization [31]. Extreme caution must be exercised in patients on long-term aspirin, because of a risk of hemorrhagic transformation [31]. Clopidogrel should be used in patients who cannot tolerate aspirin [31]. Cilostazol, another antiplatelet drug, can be used as an option. A study was conducted to compare the efficacy of clopidogrel and cilostazol in improving neuropsychological test scores. In non-surgical adults with ischemic MMD, cilostazol was associated with better cognition improvement than clopidogrel [37]. Anti-platelet drugs are not recommended for asymptomatic adults due to an inherent risk of developing an ICH in almost half of the patients [31].

Surgical management

Surgical treatment revolves around direct, indirect, and combined (direct plus indirect) revascularization procedures [1]. Surgery is beneficial over conservative management in reducing the recurrence of stroke [1,38]. In children, MMD takes a rapid course and has a poor prognosis. Therefore, surgical revascularization is recommended in MMD at a young age [39].

Direct Revascularization

A prime example of a direct revascularization procedure is the superficial temporal artery (STA) - MCA bypass. STA-ACA or occipital artery-PCA anastomosis can be used as well depending on the area of ischemia [1]. A recent meta-analysis established the superiority of direct over indirect revascularization in reducing the risk of stroke [40]. Conventionally, revascularization procedures were only used for ischemic MMD because of a lack of studies on revascularization procedures done in hemorrhagic MMD [29]. However, Japan Adult Moyamoya (JAM) trial reported its findings which proved that direct bypass reduced rebleeding in hemorrhagic MMD too. The trial documented a significant difference between non-surgical and surgical patients, based on Kaplan-Meier analysis [41]. STA-MCA bypass also relieves severe headaches in patients with ischemic MMD, by increasing the CBF [42].

One of the most common complications after direct bypass is hyperperfusion syndrome (HS) which is characterized by an increased CBF leading to neurological complications like ICH and neurological deficits [43]. SPECT is the gold standard investigation for diagnosis of HS. MRI, CT and transcranial cerebral doppler can also be used for diagnosis [43]. Treatment involves adequate blood pressure control along with control of brain edema [43]. Minocycline, by blocking MMP-9 (responsible for edema after cerebral ischemia-reperfusion), has proven to be an effective treatment for HS [44].

Indirect revascularization

Indirect surgical procedures are centered on the principle that part of a vascularized tissue when put over the brain surface can lead to the formation of new blood vessels [1]. Different methods have been described- encephalo-myo-synangiosis (EMS), encephalo-duro-arterio-synangiosis (EDAS), multiple burr-hole surgery technique, encephalo-duro-arterio-myo-synangiosis (EDAMS), and encephalo-duro-myo-arterio-pericranio-synangiosis (EDMAPS) [29]. Indirect revascularization depends on the formation of new vessels, which can take months to form. It is also dependant on age, with children showing better results than adults [45].

Combination revascularization procedures combine both direct and indirect revascularization. Direct bypass provides immediate cerebral perfusion, whereas the indirect component leads to a sustained revascularization response [46]. In the perioperative period, normocapnia, adequate body fluid balance, and blood pressure should be maintained to avoid ischemic complications [31]. Hypercapnia can lead to vessel constriction and hypocapnia via vasodilation results in steal phenomenon. This is how both hypercapnia and hypocapnia can lead to decreased CBF. Therefore, it is of utmost importance to maintain normocapnia in the perioperative period [29]. In the postoperative phase, PET/SPECT can be used to measure CBF and help in assessing the outcome of surgery [31].

*Prognosis*

Numerous factors determine prognosis. A large prospective study was conducted on adults with MMD who were managed conservatively. It reported three independent poor prognostic factors - Asian origin, history of TIAs, and decreased cerebrovascular reserve [47]. In children, due to a greater prevalence of cerebral infarction, the prognosis remains poor. In adults, the involvement of PCA in ischemic episodes and rebleeding in hemorrhagic presentation signify a dismal prognosis [29]. JAM trial has shown the effect of direct bypass in reducing rebleeding in hemorrhagic MMD, thereby improving the prognosis [41]. A meta-analysis was done to assess the impact of surgery on the long-term functional outcomes in patients with MMD. It reported a good functional outcome rate of 82% based on Kim’s category. According to the meta-analysis, combined surgery had better outcomes than the indirect revascularization procedures [48].

## Conclusions

MMD is a cerebrovascular occlusive disease with the potential to cause stroke, epilepsy, and neurological deficits in adults and children alike. Chromosome 17 (RNF213) plays a part in pathogenesis but the exact molecular mechanism remains to be confirmed. The development of a genetic repository of the disease has been slow and well-founded studies for gene discovery are much needed. Angiography study is necessary to make a diagnosis. Direct and indirect revascularization procedures are used to prevent recurrence of symptoms in both ischemic and hemorrhagic MMD. Combination procedures continue to gain popularity because they provide immediate reperfusion of the cerebrum along with sustained revascularization. Further research is needed to establish the role of biochemical markers like bFGF in assessing the status of angiogenesis post-revascularization. Despite the advancements made in medical and surgical management over the past 6 decades, the prognosis of MMD is still not very favorable.
